# Abnormal Base Excision Repair at Trinucleotide Repeats Associated with Diseases: A Tissue-Selective Mechanism 

**DOI:** 10.3390/genes4030375

**Published:** 2013-07-25

**Authors:** Agathi-Vasiliki Goula, Karine Merienne

**Affiliations:** Programme of Translational Medicine and Neurogenetics, Institute of Genetics and Molecular and Cellular Biology (IGBMC), UMR 7104-CNRS/INSERM/Uds, 1 rue Laurent Fries, 67404 Illkirch, France; E-Mail: agathi.goula@hotmail.fr

**Keywords:** trinucleotide repeat diseases, instability, BER

## Abstract

More than fifteen genetic diseases, including Huntington’s disease, myotonic dystrophy 1, fragile X syndrome and Friedreich ataxia, are caused by the aberrant expansion of a trinucleotide repeat. The mutation is unstable and further expands in specific cells or tissues with time, which can accelerate disease progression. DNA damage and base excision repair (BER) are involved in repeat instability and might contribute to the tissue selectivity of the process. In this review, we will discuss the mechanisms of trinucleotide repeat instability, focusing more specifically on the role of BER.

## 1. Trinucleotide Repeat Instability in Diseases

Trinucleotide repeat (TNR) disorders define a group of more than fifteen neurodegenerative, neurological and neuromuscular diseases [1,2]. These genetic diseases result from the aberrant expansion of TNRs within specific genes. Various types of TNRs can cause diseases, including CAG repeats (Huntington’s disease, HD, and several dominant spinocerebellar ataxia), CTG repeats (myotonic dystrophy 1, DM1), CGG repeats (fragile X syndrome, FXS) and GAA repeats (Friedreich ataxia, FRDA). Noticeably, CAG/CTG repeat diseases are the most frequent, accounting for a dozen of disorders. TNRs are polymorphic in the control population; however, above a threshold of 30 to 50 repeat units, the repeats are pathogenic. TNRs associated with diseases are found in various parts of genes, including 3' and 5' UTRs, exons and introns. The location of the repeat expansion within genes influences the pathomechanism, which ranges from loss-of-function (FXS, FRDA) and protein and RNA gain-of-functions (HD and DM1, respectively). 

TNR expansions are dynamic mutations, which are ongoing across generations and within tissues, due to germline and somatic instability, respectively [3]. The outcome of repeat instability is contraction or expansion of the repeat. Expansion in the germline can lead to an anticipation effect, corresponding to worsening of the disease in successive generations, *i.e.*, earlier onset and more rapid progression of the symptoms [4]. Depending on the disease, a parent-of-origin effect is observed, resulting in paternal or maternal bias of expansion or contraction. In somatic tissues, expansion events tend to be more frequent than contraction events, leading to the progressive increase of the repeat tract with age. Importantly, somatic instability is tissue-selective. In most TNR diseases, the degree of variation of the repeat tract over time is highly dependent upon tissue or cell-type, and the tissues or cells presenting high TNR instability vary to some extent between diseases [2]. In HD and DM1, somatic instability is most prevalent in the affected tissues and has been proposed to act as a disease modifier, accelerating disease progression [5,6,7,8]. In HD, CAG instability is most elevated in the striatum, the tissue that is most affected [6,9,10,11,12,13]. It is noteworthy that in most CAG/CTG diseases, repeat instability is generally elevated in brain tissues, with the exception of the cerebellum, which presents limited repeat instability [2]. Interestingly, somatic CAG instability is usually great in the central nervous system and, more specifically, in neurons, indicative of the implication of replication-independent mechanisms [6].

## 2. Trinucleotide Repeat Instability as the Result of Erroneous DNA Repair

Mechanistic models of TNR instability are based upon the assumption that TNRs form stable DNA secondary structures, and error-prone repair of those structures results in repeat size variation [1,3,14]. *In vitro* experiments have shown that TNR sequences can adopt several structures, and the sequence of the repeat influences both the stability and the nature of the DNA structure [15,16]. For instance, slipped-out CAG and slipped-out CTG repeats adopt predominantly random coil and hairpin conformation, respectively, which explains the increased stability of CTG-associated DNA structures relative to CAG-associated DNA structures [17,18,19,20]. In addition, increasing the length of TNRs augments the stability, as well as the complexity of the secondary structures [15,16,21]. Sequences of more than 10 CAG/CTG repeats can show a pattern involving multiple loops or hairpins [21,22]. An additional level of complexity of DNA structures is suggested by a recent study showing interconverting conformations of slipped-DNA junctions formed by TNRs [23]. Finally, stable DNA:RNA hybrids (R-loops) can also form during transcription across TNRs [24,25]. 

It remains to be determined whether unusual DNA:DNA structures truly form under physiological conditions, particularly in cells or tissues presenting high levels of repeat instability. However, several pieces of indirect evidence support their *in vivo* existence. Cellular processes altering DNA or chromatin structure, including DNA repair, replication, transcription and epigenetic-related mechanisms, have been shown to contribute to TNR instability [3]. These processes would either promote the formation of secondary structures at repeats or induce their error-prone repair. 

More specifically, studies performed using mice modeling DM1, HD, spinocerebellar ataxia type 7 and spinocerebellar ataxia type 1 indicate that the chromatin, transcription and replication status at repeats modulates repeat instability through gene-specific *cis*-elements, which include CCCTC-binding factor (CTCF) sites, CpG islands and replication origins [26,27,28,29]. It would remain to be examined whether the level of trans-factors regulating *cis*-element activity, including CTCF, modulate susceptibility to instability in tissues. 

In addition, it has been shown that trans-factors involved in DNA repair are physiological modifiers of TNR instability. Specifically, in HD and/or DM1 mice, TNR instability is reduced upon inactivation of genes involved in mismatch repair (MMR), including *Msh2*, *Msh3* and *Pms2* [30,31,32,33,34], base excision repair (BER), including *Ogg1* and *Neil1* [35,36], and nucleotide excision repair (NER), including *Xpa* and *Csb* [37,38]. Thus, abnormal repair at TNR promotes instability. How these different factors and mechanisms interplay in a given tissue is unclear, but it is likely that the contribution of each is dependent upon the tissue considered. Therefore, investigating the mechanisms underlying tissue-selectivity should help in deciphering TNR instability. Our recent data suggest that BER is one mechanism involved in the tissue selectivity of CAG/CTG repeat instability. Below, we discuss, more specifically, the physiological role of BER in the TNR instability associated with disease.

## 3. *In Vivo* Mechanism of BER in Trinucleotide Repeat Instability

BER is a DNA repair pathway specialized in the elimination of DNA base damage, of which 8-oxoguanine (8-oxoG) is the most common DNA lesion [39,40]. BER is characterized by a sequence of highly coordinated steps, starting from the removal of the DNA base lesion by a DNA glycosylase. This results in the formation of an abasic site, which is cleaved by an AP endonuclease (Ape1 in mammals) [41,42]. The DNA strand break is then processed by either single-nucleotide base excision repair (SN-BER) or long-patch base excision repair (LP-BER). In SN-BER, DNA polymerase β (Polβ) incorporates a single nucleotide and incises the remaining 5'-sugar phosphate, prior to ligation by DNA ligase III (Lig3). In LP-BER, the flap endonuclease 1 (Fen1) removes the 5'-flap structure generated during the multi-nucleotide synthesis step mediated by Polβ or a replicative DNA polymerase prior to ligation by DNA ligase I (Lig1) [41,42]. 

### 3.1. BER in Various Models of Trinucleotide Repeat Instability

Yeast studies demonstrated the first evidence that allowed insight into the involvement of BER proteins in TNR instability. Deficiency or haploinsufficiency of *rad27*, the homolog of mammalian Fen1 in yeast, led to length-dependent CAG/CTG expansion and instability [43,44]. On the other side, it is the overexpression of Lig1 homolog in yeast (*cdc9*) that yielded longer repeat tracts [45]. Overexpression of an inactive form of cdc9 possessing a functional binding site for proliferating cell nuclear antigen (PCNA) led to similar results, suggesting that instability is dependent upon PCNA interaction rather than DNA ligase activity [45,46]. Mutations in PCNA and the replicative DNA polymerase Polδ also induced destabilization of the repeat tract [47]. Fen1, PCNA, Lig1 and the replicative DNA polymerases are involved in both replication and LP-BER, and yeast studies did not allow discriminating of whether replication and/or LP-BER contribute most to TNR instability. The effects of Lig1 protein level, activity and capacity to interact with PCNA on replication and repair at CAG/CTG repeats were assessed using human cells and plasmid-based substrates [48]. Consistent with the yeast studies, disruption of Lig1 and PCNA interaction increased instability, due to errors during replication, whereas Lig1 overexpression increased repair-dependent TNR instability. In addition, reduced Lig1 activity did not alter instability.

Mouse genetics was further used to attempt to define the role of BER proteins in TNR instability. DM1 mice were crossed with knock-in mice carrying a mutation in Lig1, resulting in very low residual ligase activity [49,50]. Somatic CTG/CAG instability in DM1 mice expressing the Lig1 mutant was similar to that in DM1 mice. However, DM1 mice mutants for Lig1 showed a maternal instability bias, leading to increased contractions and decreased expansions. Furthermore, HD and DM1 mice were crossed with mice haploinsufficient for *Fen1*. Somatic CAG/CTG instability was unchanged in HD and DM1 mice heterozygous for *Fen1*, regardless of the tissues analyzed, though a modest effect on germline instability was observed in HD mice haploinsufficient for *Fen1* [51,52]. These results might indicate that the mechanisms underlying CAG/CTG instability in yeast and mammals are different. The involvement of replication in repeat instability might be prominent in proliferative cells, such as yeast, but more limited in mammalian tissues. Alternatively, different compensatory mechanisms might take place in yeast and mammalian tissues that could account for the different effects induced by deficiency of Fen1 or Lig1 in the two model systems. Of note, in contrast to yeast, the complete inactivation of *Fen1* could not be achieved in mice, due to the embryonic lethality of full knock-outs [53,54]. In general, assessing the role of BER proteins in CAG/CTG instability using mouse genetics is a difficult task, as inactivation of the main BER genes, including *Polβ* [55,56], *Ape1* [57], *Xrcc1* [58], *Fen1* or *Lig1*, is embryonically lethal.

DNA glycosylases represent an exception to this rule. The inactivation of individual DNA glycosylases is compatible with life in mammals, due to functional redundancy. Interestingly, full inactivation of the DNA glycosylase, *Ogg1*, in HD mice led to reduction of the age-dependent repeat instability in the brain and liver, suggesting the repair of 8-oxoG lesions promotes somatic CAG instability [35]. Inactivation of *Ogg1* or *Ape1* in a human cell model allowing for detection of repeat contraction events only did not improve repeat stability, possibly due to the low frequency rate of contraction in this model system [59]. In addition, HD mice deficient for the DNA glycosylases, *Aag* and *Nth1*, which remove alkylated purines and pyrimidine-derived lesions, respectively, had no effect on CAG instability [35]. In addition, somatic and germline instability was reduced in HD mice deficient for *Neil1*. Interestingly, somatic instability was decreased in all tissues tested, which included brain and non-brain tissues. Neil1 is a DNA glycosylase that targets pyrimidine-derived lesions, like NTH1 [36]. However, Neil1 can remove DNA lesions in both duplex and single-strand DNA (ssDNA), and can also remove 8-oxoG lesions in both DNA configurations, though removal efficiency for this DNA lesion is poor [60]. Importantly, CAG instability in HD mice was only moderately reduced upon inactivation of *Ogg1* and *Neil1*, and phenotype penetrance (e.g., improved CAG instability) was partial, suggesting that neither Ogg1 nor Neil1 is essential regarding CAG instability, possibly due to overlapping substrate specificities. Whereas these data demonstrate that DNA glycosylases contribute to CAG instability *in vivo*, the exact nature of mutagenic DNA lesions remains elusive. Assessing the effect of inactivation of additional DNA glycosylases could help in answering this question. Finally, it remains to be determined whether DNA glycosylases contribute to repeat instability in other models of TNR diseases. Interestingly, it was reported that the DNA oxidizing agent, potassium bromate, exacerbates germline repeat expansion in a fragile X premutation model, suggesting that repair of oxidative DNA lesions might be involved in the instability of CGG repeats [61].

### 3.2. Level and Accessibility of DNA Lesions at Trinucleotide Repeats

Accumulation of oxidative DNA damage with age in specific tissues could explain the age dependency and tissue specificity of somatic instability [35]. However, though the global level of DNA damage increased with age in HD mouse striatum and cerebellum, it was lower in the striatum, which presents high CAG instability levels, when compared to the cerebellum, showing minimal repeat instability [62]. In addition, DNA damage at CAG repeats did not increase with age and was not higher in striatum, though it was abnormally high in HD mouse tissues [62]. These data suggest that the amount of DNA lesions at CAG repeats does not directly contribute to age-dependent and tissue-selective somatic instability, suggesting that other mechanisms explain these features. 

The increased level of DNA lesions at CAG repeats found in tissues of HD mice might result from their reduced accessibility to DNA repair proteins. Several studies support this hypothesis. Repair of hairpin-forming substrates with DNA lesions, including 8-oxoG, 5-OHC and the AP site, was impeded, likely due to reduced binding of DNA repair proteins to hairpin structures [36,62,63,64]. Along this line, the degree of stiffness of CAG/CTG substrates with slip-outs negatively influenced repair efficiency [23]. Additionally, hairpin substrates with CAG/CTG repeats contained a hot spot for DNA damage [63]. Thus, both reduced accessibility and increased susceptibility to DNA damage could contribute to lesion accumulation at CAG/CTG expansions.

### 3.3. Mechanism of BER in Tissue-Selective CAG/CTG Repeat Instability

Several *in vitro* and cell-based studies have provided insights into the mechanism by which BER proteins might contribute to CAG/CTG instability. It has been reported that processing of CAG/CTG substrates by Polβ, a central component of BER, leads to strand displacement and multi-nucleotide gap filling, due to polymerase slippage at repeats [65,66]. Polymerase slippage would induce the formation of a 5'-flap structure, which would require LP-BER proteins, including Fen1 and Lig1, for completion of repair. Since stable secondary structures forming at TNR repeats prevent efficient excision by Fen1 [67,68], it was hypothesized that expansion of the repeat tract could arise from inefficient excision or alternate cleavage by Fen1 of the 5'-flap structure generated upon multi-nucleotide gap filling by Polβ, followed by ligation of an erroneous number of repeats [14,66]. Alternate cleavage by Fen1 would be required for the generation of a ligatable nick and completion of repair. Studies using partially and fully reconstituted repair assays support these possibilities and further indicate that the level of coordination of BER enzymatic steps is critical in determining the repair outcome at CAG/CTG repeats [69,70,71]. The processing of CAG/CTG substrates preferentially involved LP-BER in contrast to substrates with a random sequence, suggesting that LP-BER is required to repair a lesion at CAG/CTG repeats. Furthermore, repair outcome was influenced by the stoichiometry of BER proteins [62,71]. BER protein levels and activities greatly varied between the striatum and the cerebellum. Although the level of Polβ was similar in the two tissues, LP-BER proteins, including Fen1 and Lig1, were much more abundant in the cerebellum as compared to the striatum. As a result, repair efficiency at CAG/CTG repeats was poor and led to the formation of persistent intermediate products when using the striatal BER protein stoichiometry, as compared to that of the cerebellum. It was hypothesized that the sub-optimal BER activity in the striatum contributes to the increased striatal instability seen in HD, whereas the efficient and well-coordinated BER activity in the cerebellum might limit instability in this tissue, preventing the formation of secondary DNA structures at repeats.

**Figure 1:**
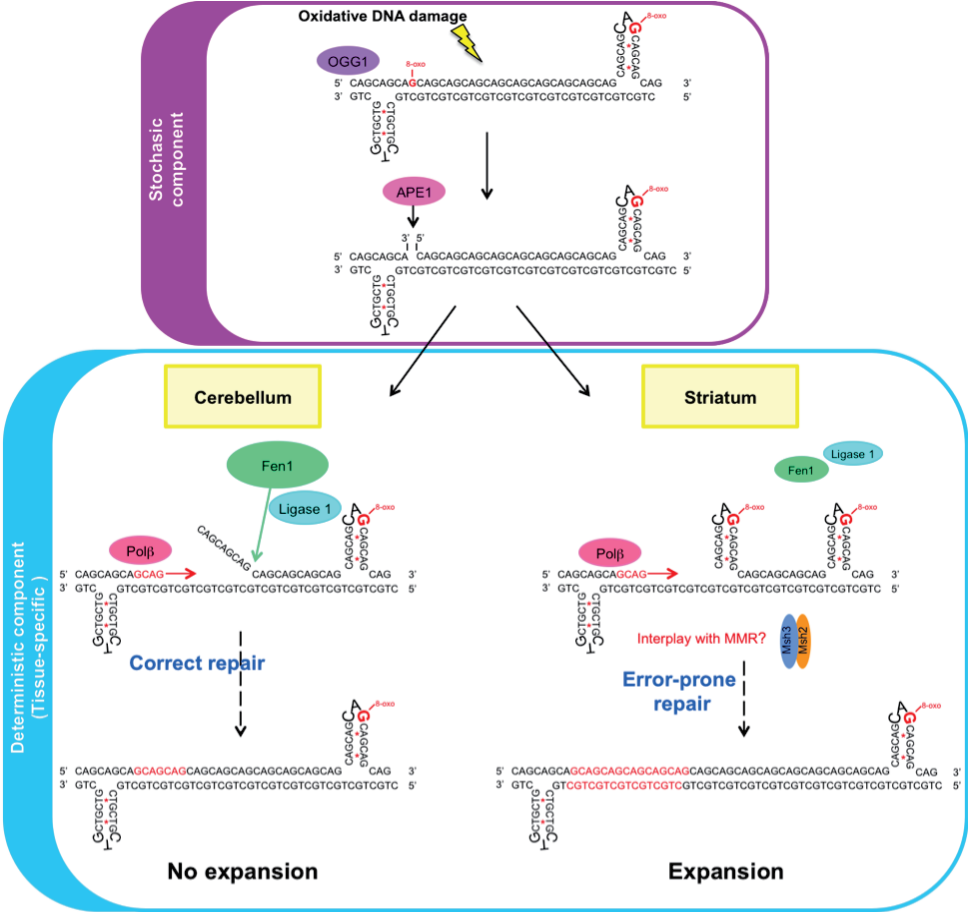
Base excision repair (BER)-induced CAG repeat expansion is tissue-dependent. Oxidative DNA lesions, including 8-oxoG lesions, occur stochastically at trinucleotide CAG repeats and are processed by the BER pathway. A DNA glycosylase (e.g., Ogg1) and Ape1 initiate repair. Repair outcome (“no expansion” or “expansion”) is dependent upon the location of the lesion and the tissue that is repaired. In the cerebellum, where Fen1 and Lig1 are abundant, the DNA lesion at CAG repeats is correctly repaired: the flappy structure resulting from multinucleotide incorporation by Polβ during long-patch (LP)-BER by Fen1 is efficiently processed, and the subsequent ligation step does not result in expansion. In contrast, in the striatum, where Fen1 and Lig1 proteins are reduced, repair of the DNA lesion at CAG repeats is error-prone. The flappy structure is not efficiently processed, which ultimately leads to repeat expansion through a yet unknown mechanism. Additional DNA repair pathways, including mismatch repair (MMR), might interplay with BER during this process.

Moreover, the sequence of the repeat (CAG *versus* CTG) and the position of the lesion within the CAG/CTG substrates also influenced repair outcome, e.g., repair efficiency and LP-BER requirement [71,72]. The results by Lai *et al.* suggest that the position of the lesion within the repeat substrate determines whether expansion or contraction occurs [72]. It was reported that a lesion located at the 5'-end of CTG repeats results in expansion, whereas a lesion located in the middle or the 3'-end of the repeats results in deletion. In studies using plasmid-based CAG/CTG substrates and mammalian cell extracts, it was also reported that the position of a nick with respect to the repeat tract influences repair outcome [18,20,22,73]. In these assays, repair efficiency was decreased when the slip-out was located on the CTG strand in comparison to the CAG strand. The mechanism of TNR instability includes both stochastic and deterministic components [74]. The occurrence, accessibility and location of a DNA lesion at repeats are stochastic events that might contribute to the stochastic component of instability, whereas damage repair would be involved in the deterministic component. Since BER protein stoichiometry varies between tissues, this BER deterministic component might be tissue-dependent (Figure 1). Interestingly, the level of MMR proteins, including Msh2, Msh3 and Msh6, is also highly variable between mouse tissues [75]. It was suggested that the elevated levels of MMR proteins in embryonic stem cells of DM1 patients might contribute to the high CTG instability level found in these cells [76]. Similar conclusions were drawn using induced pluripotent stem cells derived from fibroblasts of Friedreich ataxia patients [77,78]. However, the relative levels of MMR proteins were higher in the cerebellum, as compared to the striatum and cortex, indicating that high MMR protein amounts do not correlate with high instability levels in somatic tissues. Further studies are required to specify how tissue-specific regulation of trans-factors impacts on tissue selective instability.

## 4. Conclusions

Many genetic diseases are caused by dynamic mutations, including trinucleotide repeat expansion diseases. Repeat instability can accelerate disease progression. Understanding the etiology of TNR instability is crucial, since instability might represent a therapeutic target. During the last decade, important advances have been made that increase our understanding of the rules governing this particular type of instability. Specifically, it was discovered that oxidative DNA damage and BER play a physiological role in the somatic CAG instability involved in HD and possibly contribute to the tissue selectivity and stochasticity of the instability process. Yet, how broad the involvement of oxidative DNA damage and BER in the TNR instability associated with diseases is would need to be investigated. How BER interacts with other mechanisms modulating TNR instability, including MMR, transcription and replication, is also a question that would deserve interest.
